# Reconstruction of forest dynamics in the Western Palaearctic based on phylogeographic analysis of the ringlet butterfly *Erebia aethiops*

**DOI:** 10.1038/s41598-020-79376-x

**Published:** 2021-01-08

**Authors:** Martin Wendt, Martin Husemann, Katja Kramp, Thomas Schmitt

**Affiliations:** 1 Senckenberg Deutsches Entomologisches Institut, Systematik Und Biogeographie, Eberswalder Str. 90, 15374 Müncheberg, Germany; 2 Centrum Für Naturkunde, Universität Hamburg, Martin-Luther-King-Platz 3, 20146 Hamburg, Germany; 3 Leibniz-Zentrum Für Agrarlandschaftsforschung (ZALF) E.V., PB 2: „Landnutzung Und Governance“, AG: Biotische Interaktionen Zwischen Wald- Und Agrarflächen, Eberswalder Str. 84, 15374 Müncheberg, Germany; 4 Zoology, Institute of Biology, Faculty of Natural Sciences I, Martin Luther University Halle-Wittenberg, 06099 Halle (Saale), Germany

**Keywords:** Biogeography, Climate-change ecology, Forest ecology, Entomology, Population dynamics

## Abstract

Glacial refugia are centers of high biodiversity. Therefore, knowledge on their locations and reactions of associated populations and landscapes to climatic changes is crucial for conservation management. We here investigated the biogeography of a butterfly species linked to open forest habitats. Using mitochondrial and nuclear markers in combination with Bayesian simulations, we analyzed the location and age of potential glacial refugia of the species. We identified five putative refugia in Europe. Considering the ecological needs of our study species, tree density within these refugial areas, in contrast to earlier assumptions, must have exceeded the level of individually scattered trees. Our results also provide evidence that especially the refuge areas in the Carpathians were previously underestimated regarding their age: the refugia in the Southern Carpathians presented suitable conditions throughout several glacial cycles, probably since the Mindel or Riss cycles. Additionally, our analyses provided support for a forest refugium near the Tatra Mountains persisting the last glacial maximum. Our results underline the usefulness of this and probably other butterfly species as indicators of forest refugia.

## Introduction

Distributions of living organisms are subject to permanent change with glacial and interglacial cycles of the Pleistocene having strong effects on the ranges of animals and plants worldwide, especially in Europe^1^. During cold stages, warm-adapted species were restricted to refugia. These species in general retreated towards the equator with the onset of a glaciation; consequently, the Mediterranean peninsulas became important centers of survival and re-colonisation in the western Palearctic^2–4^. Various biogeographical studies extended the knowledge about glacial refugia and added Arctic-Alpine and Siberian faunal elements for Europe^5^. Genetic studies discovered numerous genetic lineages, often with complex structures, and thus led to the exploration of extra-Mediterranean and cryptic refugia in Europe^6^.

Meanwhile, it has been shown for a larger number of temperate species representing a large array of different animal and plant groups that they have survived glacial conditions in northern, often cryptic refugia^7–9^. In a number of cases, species occupied both classical Mediterranean and extra-Mediterranean refugia^10–13^. The majority of these species are those of open land or semi-open landscapes. However, species ecologically linked to closed forest structures so far are rarely detected for these northern refugia. Nevertheless, numerous tree species have survived the last cold phase of the Pleistocene in these extra-Mediterranean refugia, according to studies using subfossil evidence of timber, pollen profiles, genetic analyses, and niche modeling^14–16^. These lines of evidence demonstrate that retreat areas for tree species were located especially at the edge of high mountain systems (i.e. Alps, Pyrenees, Carpathians). In particular, the eastern edge of the Alps, the southern slopes of the Southern Carpathians, and parts of the Carpathian Basin appear to have provided suitable conditions for the survival of temperate tree species^8,17,18^. Also on the Balkan Peninsula, trees were probably more widespread under glacial climatic conditions than previously assumed^15^.

All this has significantly changed our understanding of the conditions in Pleistocene Europe. However, most studies have focused on the presence and density of single trees species in an area at a certain time, but not whether they also created the ecological conditions of a forest ecosystem^14,16^. To investigate this question, it is necessary to study species that are dependent on the existence of forest ecosystems. One such species that cannot survive without the existence of forest ecosystems is the Scotch Argus, *Erebia aethiops* (Esper, [1777])^19–23^. This satyrid butterfly is widespread in the western Palearctic in forested areas and can be found mostly continuously from the French Massif Central through Europe and western Siberia to the western Altai Mountains. In the north, it reaches the plains of northern central Europe only sporadically; in the south, it is missing in Iberia, and the entire south of the Balkan Peninsula. Relatively few populations are known from peninsular Italy. All over this range, the morphological differentiation is weak^23^; nevertheless, several morphology-based subspecies have been described, e.g. the southern Alps subspecies *E. aethiops rubia* Fruhstorfer, [1909]. However, many of these morphological lines were synonymized by Varga^24^. Although *E. aethiops* does not reach the Atlantic Ocean, an isolated population exists in Scotland, formerly described as subspecies *caledonia* Verity, [1911]. Further populations isolated from the main distribution area exist in the mountains of northern Turkey and in the Caucasus, which are currently treated as a separate subspecies, *E. aethiops melusina* Herrich-Schäffer, [1847].

To better understand the dynamics of forest ecosystems in Europe during the sequence of several glacial-interglacial cycles, we studied the phylogeography of *E. aethiops* across large parts of the species’ range from its westernmost parts in the Massif Central in France to the eastern Carpathians and the eastern Balkan Peninsula. We supplemented our dataset with available sequences from Genbank and BOLD to account for potential northern and Asian lineages and compared this entire data set with sequence data from other closely related species of the *aethiops*-group restricted to eastern Asia. We used mitochondrial and nuclear genetic information to estimate divergence times of the main differentiation events of this butterfly depending on forests to address the following questions:

1. Where did *E. aethiops* originate from and what does this tell us about the persistence of forest ecosystems in Europe?
2. Which historical processes can explain the current distribution of *E. aethiops* and which conclusions can be drawn from this for the dynamics of forest ecosystems?
3. Where were glacial refugia of *E. aethiops* located and which new insights on the dynamics of forest ecosystems can be derived from this?

## Results

### Mitochondrial DNA

Concatenated COI and NDI sequences (1,212 bp) of 133 specimens from 28 populations yielded 34 haplotypes (see genetic diversity parameters in Table 1). The most common ones were H9 (24.8%), H7 (9.0%), and H1 (8.3%); all other haplotypes had frequencies less than 4%. The maximum p-distance among *E. aethiops* haplotypes was 0.0091 (Spiazzi vs. Cheile Butii; Spiazzi vs. Nanos) with an overall mean genetic distance of 0.004 (s = 0.002).

**Table 1:** Genetic diversity parameters of the two separated and combined mitochondrial DNA markers of *E. aethiops.*

	COI	NDI	Aligned haplotypes
Nucleotide diversity Pi	0.0026	0.0028	0.0039
Haplotype diversity h	0.822	0.696	0.998
Segregation sites S	21	14	35
Average number of nucleotide differences k	1.7141	1.5733	4.7344

**Figure 1:**
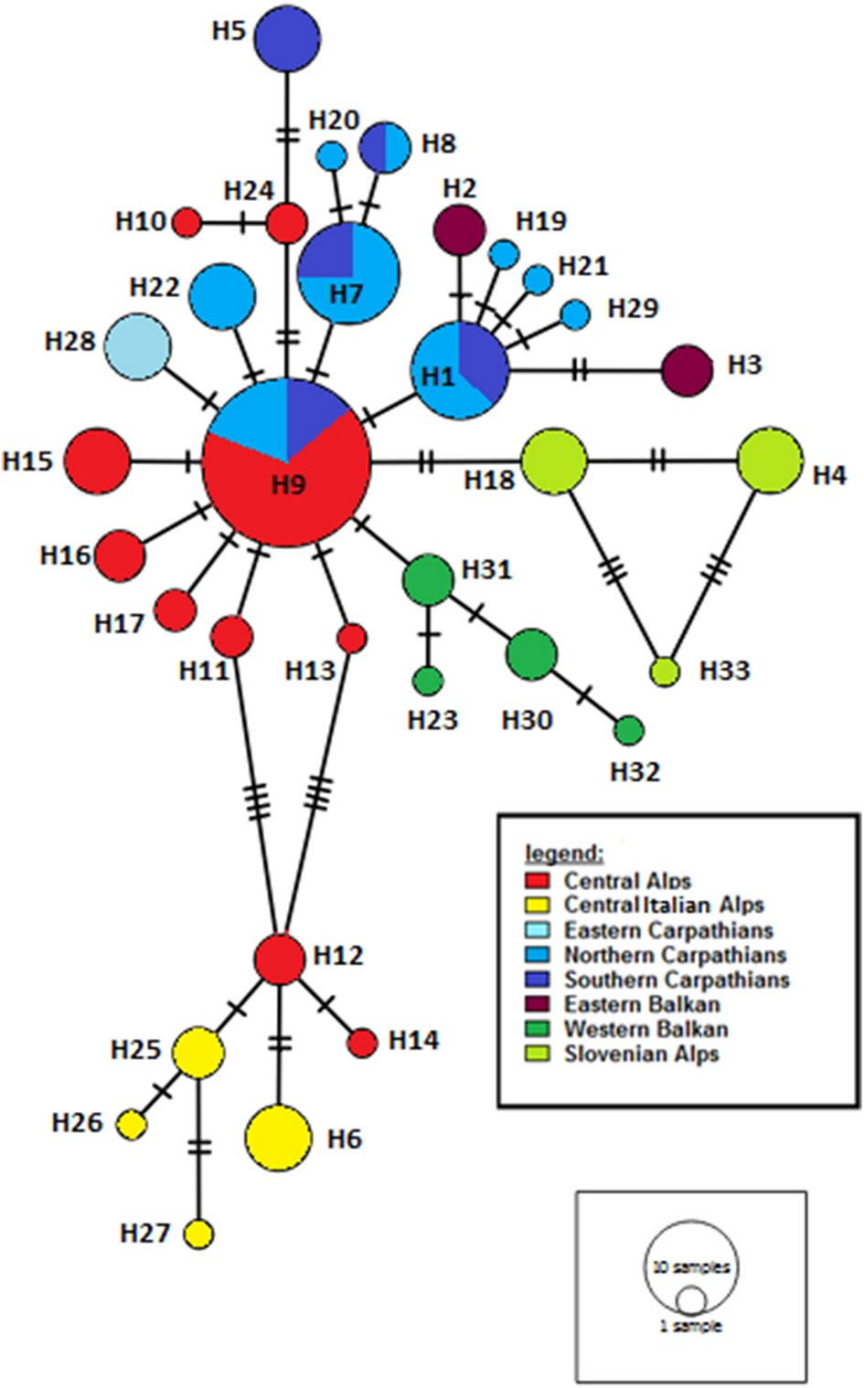
Minimum spanning haplotype network based on the concatenated mtDNA haplotypes (COI, NDI) of *E. aethiops.* Mutation steps are shown by the number of hatch marks.

Two main groups were distinguished by the haplotype network based on both mtDNA markers; a southern Alps group, and all other populations (see Fig. 1). The Central Italian Alps group was represented by two Italian populations and haplotypes detected at Plöckenpass (on the Austrian-Italian border, and thus north of the two other populations of the Southern Alps group). This group roughly coincides with the geographic distribution of *E. aethiops rubria*. Plöckenpass exhibited a mixture of haplotypes from both main groups. While no clear structure was visible in the southern Alps group, the second main group displayed a star-like pattern, with the dominant haplotype H9 being central (found in the Alps, in populations in the Tatras, and the Romanian Carpathians), and the majority of its satellite haplotypes being restricted to the Alps. In the Tatras, another group formed a secondary star-like structure with H1 at its core. However, H1 was also found in Baile Herculane (Romania) and Trigrad (Bulgaria); the latter was dominated by two satellite haplotypes of H1. The common H7 with its two satellites was also restricted to the Tatras and the Romanian Carpathians. Only the haplotypes H5 (Cheile Butii, Southern Carpathians) and H28 (Gheorgheni, Eastern Carpathians) were exclusive for the Romanian Carpathians. The three populations sampled in Slovenia had three endemic haplotypes, that were most closely related to each other. This result partially coincided with the results of the STRUCTURE analysis of allozyme data (see below) in the divergence of the Nanos population, but was contradictory for the two populations from the Slovenian Alps (Medvodje and Sija), which, according to the allozyme data, formed a group together with the large majority of populations from the Alps (see Fig. 4). The western Balkan populations of Tresnjevik, Ropojan Valley and Valbona (all of these were not represented in the allozyme analysis) had four closely related haplotypes which were geographically restricted to this region, but were linked via H31 by just one mutational step to the most common haplotype H9.

**Figure 2:**
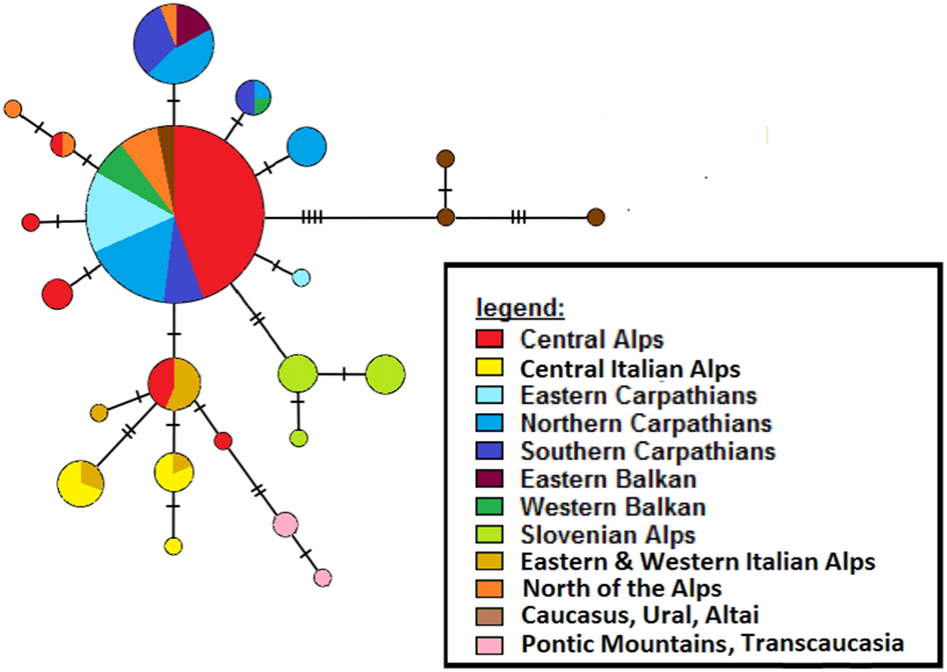
Minimum spanning Haplotype network of *E. aethiops* based on a COI fragment (429 bp). Mutational steps are indicated by the number of hatch marks.

The geographically expanded dataset with additional samples from Genbank and BOLD, based exclusively on a 429 bp COI fragment, yielded a considerably simpler haplotype network (see Fig. 2). Nonetheless, the southern Alps and the Dinaric Mountains still displayed distinct groups, whereas the western Balkans and the Carpathian region also represented the most common haplotype or direct derivates. This also applied to Germany, Scotland, and Latvia. Haplotypes from eastern Europe and Asia partly showed remarkable differentiation from the above-mentioned haplotypes. Thus, a separate lineage was located in the Pontic Mountains (northern Turkey). Despite geographic proximity and inclusion in the same morphological subspecies (i.e. *E. aethiops melusina*), samples from the western Caucasus represented a different group which genetically is most closely related to individuals from the Urals and the Altai Mountains.

**Figure 3:**
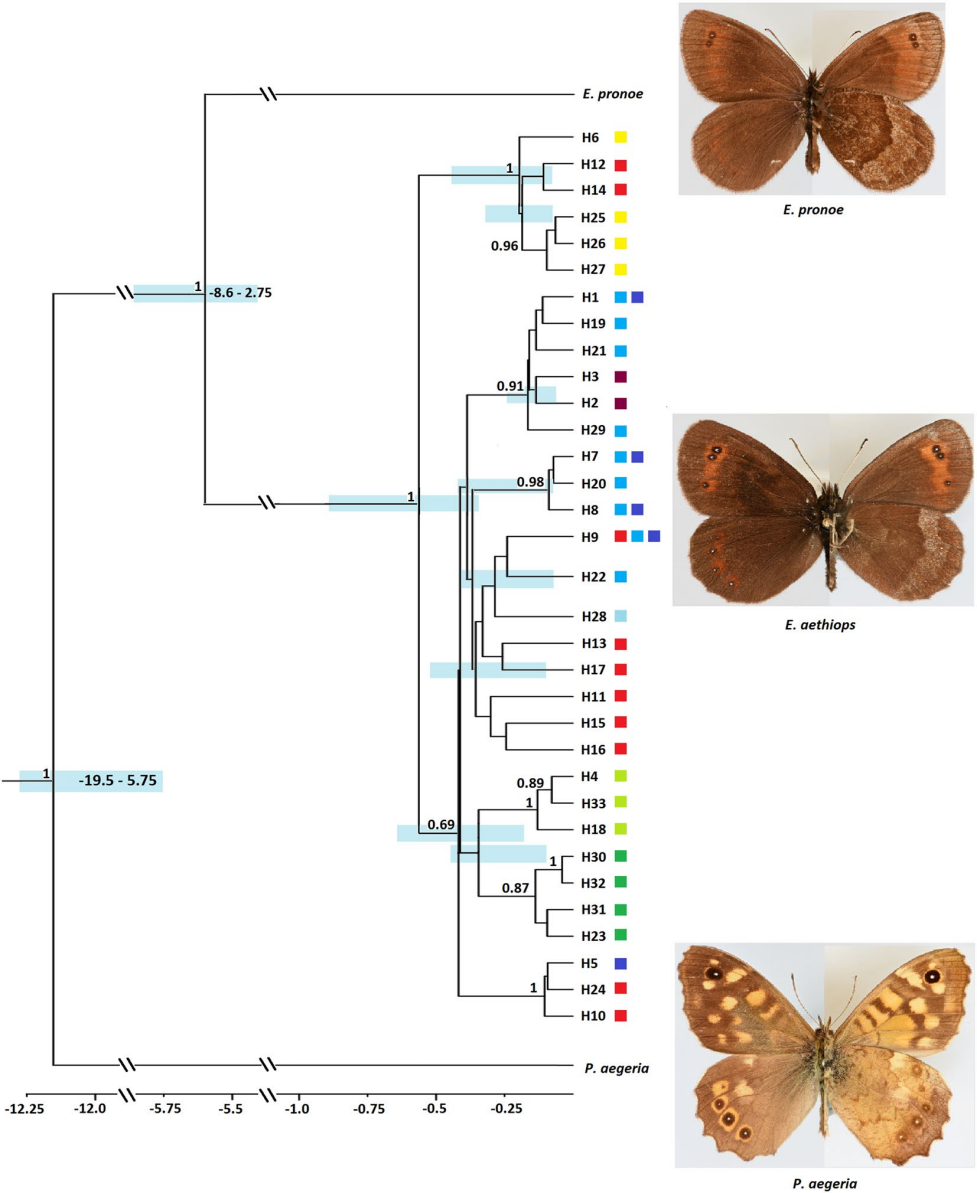
Bayesian phylogeny based on concatenated mtDNA haplotypes (COI, NDI) of *E. aethiops*. Number above recovered nodes: Bayesian posterior probabilities > 0.7; node bars: 95% highest posterior density in light blue bars. The geographical location of the haplotypes is given in same color scheme as in Fig. 1. Photos M. Wendt.

We conducted a Bayesian analysis using BEAST to date the intraspecific splits; the splitting events of the two outgroups were estimated at 5.6 mya for *E. pronoe* and 12.1 mya for *Pararge aegeria* (see Fig. 3). The southern Alps group and the widespread group split about 560 kya. The oldest split within the latter group was estimated at 420 kya separating Slovenian specimens from those of the central Alps. Three groups branched-off between 400 and 300 kya: one group representing haplotypes found in the Tatras, the Romanian Carpathians, and Bulgaria, another group with haplotypes from the Tatras and Romanian Carpathians, and the third group with haplotypes from the Alps and Cheile Butii (southern Carpathians). The split between Slovenia and the western Balkans was estimated at 400 kya. Six of the seven haplotype groups had group posterior probabilities > 85%.

Including sequence data of the other representatives of the *aethiops* group which are restricted to eastern Asia, a RASP analysis did not deliver strong support for one of the several possible ancestral areas of *E. aethiops*. A European-Asian or a solely European origin is equally likely. However, support is given for an origin of the entire *aethiops* group in eastern Asia (see supplementary S1 and S2). Bayesian Skyline plots indicated stable female effective population sizes for the more distant past, with a decline starting at about 700 kya (Günz glaciation) resulting in a bottleneck at around 130 kya (Eem interglacial). This was followed by an ongoing phase of expansion starting in the Eem interglacial (128 kya to 115 kya B.P.)^25^ (see supplementary S3).

### Allozymes

We assessed the genetic diversity of allozymes over major parts of Europe; we calculated the number of alleles per locus, expected (H_e_) and observed heterozygosity (H_o_), the percentage of all polymorphic loci (P_tot_) and the percentage of loci with the most common allele not exceeding 95% (P95) (see supplementary S4). The average number of alleles per locus was highest in the Apuseni Mountains, Tatras, and northern Italy. Central and western Alps populations had below-average diversities (1.62 ± 0.18 SD). Similar patterns were found for the observed heterozygosity (H_o_). All loci and populations were in HWE. The 14 significant deviations from the HWE (applying to single loci in single populations) were reduced to two after Bonferroni correction (i.e. Medvodje for PGM; Fernpass for G6PDH). A test of LD revealed deviations in six populations after Bonferroni correction (see supplementary S5). Six linkage pairs were detected in Medvodje, three linkage pairs in Nanos, and Szelcepuszta. Each of the remaining three populations displayed just one case of linkage disequilibrium.

A locus-by-locus AMOVA based on a weighted average over the 20 polymorphic loci revealed the highest amount of diversity within individuals (variance component: 0.681), followed by between population variance (variance component: 0.300; F_ST_: 0.263, *p* < 0.001), and the variance between individuals within populations (variance component: 0.159, F_IS_: 0.19). The unweighted genetic distance^26^ between all 27 populations varied from 0.015 to 0.219 with a mean of 0.062 (± 0.044 SD) (see supplementary S6). A Neighbor-Joining phenogram (NJ) based on these distances revealed four distinct groups: one group consists of the Italian populations of the central Italian Alps, a second geographically extensive group reaching from the Massif Central across the Alps to the Tatras, a third cluster consisted of the Romanian Carpathian populations; Nanos (in Slovenia) represented the fourth cluster (see supplementary S7).

The position of the Tatras group was not completely resolved. Most specimens were belonging to the extensive group, the remaining specimens to the Romanian one. Bootstrap values were weak, except for the strong support of the central Italian Alps group.

**Figure 4:**
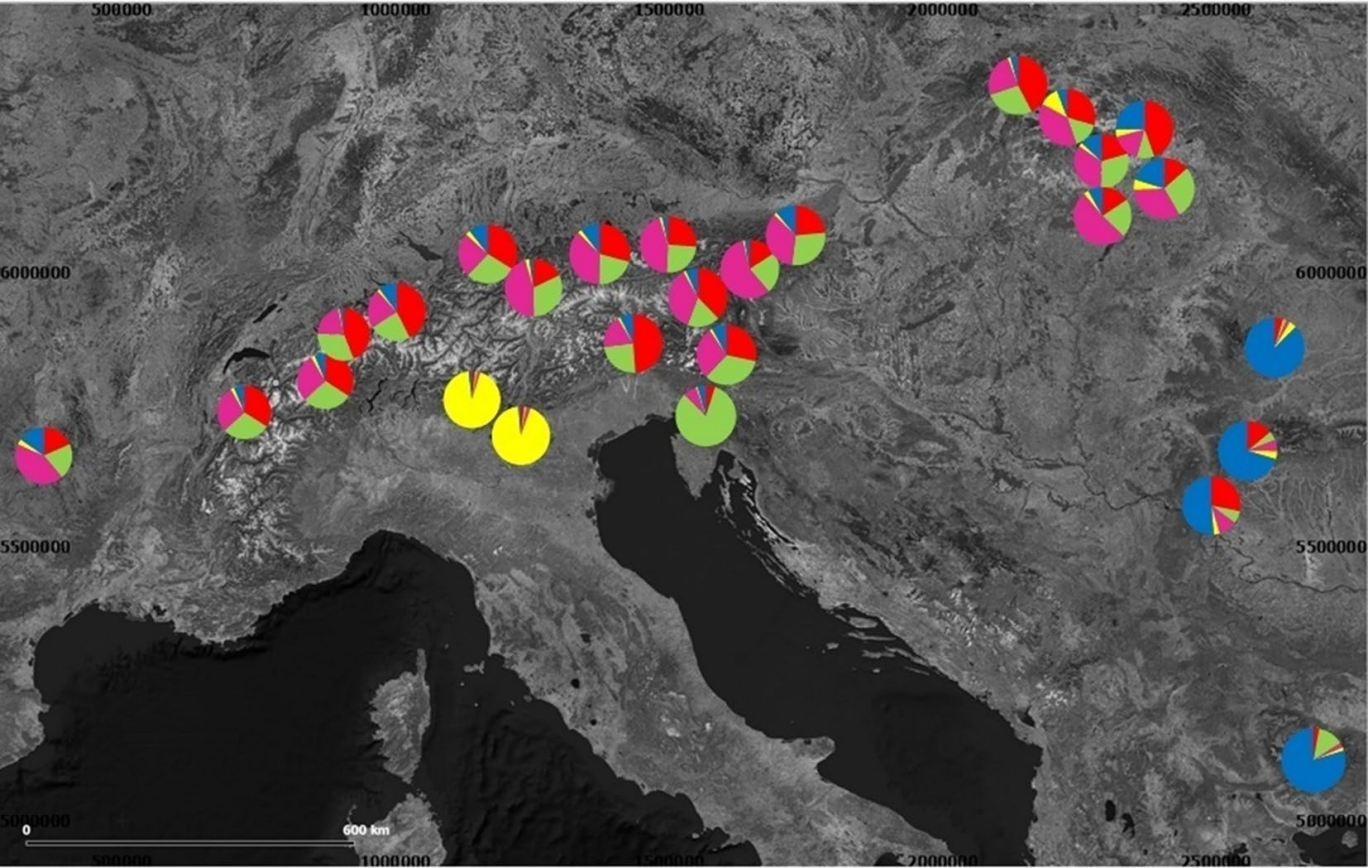
Cluster analysis with STRUCTURE for K = 5 based on allozyme polymorphism. The colors indicate the distinct genetic clusters to which the individuals of a population are assigned. The map was created with Qgis v.3.10.10^72^ (Available online: http://qgis.osgeo.org).

In addition to the NJ phenogram, a STRUCTURE analysis was conducted (see Fig. 4). The most likely ∆K value was five (see supplementary S8), since the value of two should be ignored^27^. K = 5 distinguished between four groups; the populations sampled in the central Italian Alps, the southern Carpathians and the eastern Balkans, Nanos, and a widespread group from the Massif Central to the Tatra populations, which is composed of two Structure groups (see supplementary S9). Geneland suggested three clusters, largely identical to the results of STRUCTURE (see supplementary S10, S11), yet, Nanos was grouped with the widespread group.

Pairwise genetic distances (derived for K = 5) were the highest for the central Italian Alps group (see supplementary S4), with the largest distance to the Nanos group (0.209 ± 0.014 SD) and the smallest distance to the Southern Carpathians—Eastern Balkans group (0.121 ± 0.008 SD). Pairwise F_ST_ values ranged from 0.009 (Sonnenstein vs. Medvodje) to 0.633 (Col des Aravis vs. Spiazzi) (see supplementary S12). A change of the main allele was observed in eight populations, where the highest number of three switches in the main allele was reached by Trigrad (Bulgaria) and Campolaro (central Italian Alps group). In addition, the central Italian Alps group is characterized by four endemic alleles, a number that was obtained in only one other group (i.e. the extensive group) (see supplementary S13).

## Discussion

### The center of origin of *E. aethiops* and the entire species group

The species *E. aethiops* represents a monophyletic group together with the species *E. niphonica*, *E. neriene* and *E. alcmena*^28^. As all these species with exception of *E. aethiops* are exclusively found in eastern Asia, an Asian origin of the entire species group appears to be likely. However, the region where *E. aethiops* was evolving cannot be reconstructed unambiguously. The species’ extant range stretches from western Europe to the Altai Mountains, hence allowing for several alternative areas of origin. A RASP analysis also could not point out a clear provenance (see supplementary S1). However, several aspects call for a western Palaearctic origin of the species. First, the *aethiops* group consists of five strongly supported monophyletic lineages (one per species, but two in the case of *E. niphonica*) and an unresolved backbone^28^. Apart from a limited geographic overlap between *E. neriene* and *E. aethiops* in the Altai Mountains, all lineages are distributed strictly allopatrically^28^.

Consequently, an evolution in or nearby these species’ current distribution ranges is much more parsimonious than a single speciation cradle in the Altai area with subsequent expansion of these lineages, but later extinction in their center of origin in the majority of cases. The fact that the ecological niches, with the exception of the more thermophilic *E. alcmena*, hardly differ suggest an allopatric origin instead of a common speciation origin.Furthermore, the ecologically rather similar species *E. aethiops* and *E. neriene* show an ecological character displacement in their limited area of sympatry in elevation levels in the Altai Mountains^29^, also rejecting the hypothesis of nearby centers of origin for both species. Consequently, we suggest that the origin of *E. aethiops* is west of the Altai Mountains. Such a western Palearctic origin of *E. aethiops* was already postulated by Pena et al.^30^, but based on a dataset not including its sister species analyzed by Nakatani et al.^28^.

The internal genetic structures of *E. aethiops* give additional evidence for a European origin of the species because the COI sequences reported from the Pontic Mountains and Transcaucasia (both northern Turkey) on the one hand and the ones from the Urals to the Sajan Mountains on the other are branching from two opposite sides of the European network. Additionally, the sequences of the individuals from the Russian Caucasus (i.e. north of the main ridge of these mountains) are directly derived from the Ural/Sajan Mountains group, showing its maximum p-distance towards the geographically nearby populations of Transcaucasia.

Based on these data, the following evolutionary scenario and range dynamics seem the most likely: a population group expanded from Europe to Asia Minor with subsequent allopatric differentiation in the mountain ranges of northern Turkey. An additional range expansion likely occurred along a north-eastern pathway reaching as far east as the Sajan Mountains as its eastern endpoint. This expansion has given rise to a secondary one in southerly direction, reaching as far south as the Russian Caucasus. This scenario well explains why the most genetically distinct populations of this species are in such close geographic proximity: they represent the two endpoints of an pincer-like expansion rooted in Europe. As the genetic differentiation at the Eurasian level is considerably higher than in Europe alone, the time frame of these shifts must be seen prior to the beginning of the differentiation in Europe (i.e. 560 ky BP), but due to the differentiation depth, the split likely is not older than the onset of the Pleistocene.

### The phylogeography of *E. aethiops* in Europe

When looking at Europe in more detail, the oldest split within *E. aethiops*, detected at the mitochondrial and nuclear level, is between the Central Italian Alps populations and all others. The age of this split was estimated at about 560 ky BP, i.e. approximately at the beginning of the pronounced glacial-interglacial cycles with about 100,000 years per full cycle^25^. This matches other molecular clock-based estimates of divergence events of other taxa in this region^7^. Hence, it is likely that the distribution of European *E. aethiops* was covering at least parts of the Alps and its surrounding areas prior to the onset of the Günz glacial. Due to the climatic deterioration during this ice-age, which was not as severe as the following ones, but nevertheless stronger than before^25^, this formerly continuous distribution area was apparently split up. Consequently, a previously functional belt of interconnected forest ecosystems at the edge of the Alps must have been disrupted by climatic changes for the first time in the Pleistocene. This apparently has led to the spatial separation into two large population groups of *E. aethiops* in Europe.

One of these two groups correspond to the lineage currently found in the Italian parts of the Alps adjoining the Po Valley. However, due to the lineage’s rear edge position, it did not participate in all the following range dynamics^31^. The genetic diversity patterns at the mitochondrial and nuclear levels as well as the structure of the respective part of the mitochondrial haplotype network indicate a long-lasting persistence in the areas adjoining the Po Valley at low abundance, but largely without strong fluctuations in the total number of individuals. The second (i.e. the widespread) group’s geographic centre of origin cannot be reconstructed as clearly as in the first case, but our data suggest the eastern or south-eastern part of the Alps for several reasons. The genetic diversity at the nuclear level is still highest here today, a centre west of the Central Italian Alps group would not be compatible with the southern Alps group’s rear edge status and, as known from other studies^13,32,33^, the eastern and southeastern Alps probably represent one of the most important centres for glacial survival of species dwelling in forests, next to the Illyrean region, as indicated by the endemic Slovenian and Albanian haplotypes.

All further range dynamics of the widespread *E. aethiops* lineage must have taken place from this putative eastern Alpine centre of dispersal. A molecular clock analysis suggests a largely synchronous split at about 400 ky into six lineages. These lineages are also distinguished by the haplotype network, which together with the genetic distances identifies two differentiation levels within these six lineages. Thus, two comparably older lineages are found in Slovenia and Cheile Butii, with the latter located in a deep valley system in the southern Carpathians. All other haplotypes found in the Alps, northern, eastern, and southern parts of the Carpathians, western and eastern parts of the Balkan Peninsula are representatives of the younger differentiation events.

The range expansion during the mid-Mindel interstadial, which falls approximately within the time frame at about 400 ky^34^, and their long-term biogeographical consequences cannot be reconstructed unambiguously. However, a range expansion over the Carpathian region almost certainly must have taken place. It must have been facilitated by a forested link between the north-eastern Alps and the Tatras and adjoining northwestern Carpathian areasduring interglacials^18^. This connection between these two regions, involving gene flow, is well documented when it comes to mountain species^35^, but so far is not well-known regarding species intimately linked to forests.

The persistence of *E. aethiops* in the Carpathians since then can be explained by two partly diverging lines of evidence. The haplotype tree suggests the existence of several spatially separated glacial refugia in the southern Carpathians both during the Mindel and the Riss ice age. The haplotype network with its few slightly differentiated haplotypes partly contradicts this interpretation and requires a stronger reduction of populations in the Carpathians during these periods. The differentiated haplotype H5, restricted to the southern Carpathian location Cheile Butii, supports this hypothesis. These results are in line with previous findings that prove the survival of trees in the Carpathians during glacial periods^8,14,18^, as well as the existence of extra-Mediterranean refugia of arboreal species there^6^. However, our results also indicate that the existing communities were not just habitats with single trees, but that they must have represented forest ecosystems, at least locally.

The colonisation of the western Balkan Peninsula might also go back to the Günz-Mindel interglacial, but cannot be reconstructed with certainty. Based on the haplotype tree, the expansion to this region should have taken place via Slovenia. In the subsequent glacial period, the link between the Slovenian populations and the eastern Alps should have been disrupted first, followed by the split between the western Balkans and Slovenia. However, the haplotype network and the genetic distances contradict this hypothesis and indicate independent dispersal events regarding the origin of the genetic lineages endemic to these two regions. Based on our mitochondrial data, the Slovenian populations must have become separated from those of the eastern Alps during the Mindel ice-age. This is also supported by our nuclear data. Consequently, the following range dynamics seem plausible: the forest ecosystem on the eastern and south-eastern margin of the Alps that still might have been uninterrupted during the Günz ice age must have become disconnected during the colder Mindel glacial, leading to vicariance in *E. aethiops* in this region. The high number of endemic species in Slovenia^36–38^ and several phylogeographic studies^12,39,40^ support a comparatively long isolation in Slovenia, also of *E. aethiops*. In this case, the Slovenian populations, similarly to the Central Italian Alps lineage, must have held a rear edge position, and the colonisation of the western Balkan Peninsula (at least as far south as Albania) must have occurred independently from the eastern Alpine region, without the Slovenian populations having any impact on them. Our data consequently suggests that forest ecosystems were present in Slovenia, just as in the Carpathian region, at least since the Mindel glacial, but probably throughout the entire Pleistocene. This goes beyond the previous knowledge of the uninterrupted existence of trees and arboreal species^8,41,42^. Furthermore, our data provide additional evidence for the importance of the western Balkan Peninsula as a Mediterranean glacial refugium for arboreal biota.

Looking at the phylogeographic structure of *E. aethiops* across the Balkan Peninsula, a frequently observed pattern is apparent, i.e. the separation between a western and an eastern Balkan lineage^43^. Whilst the northward range expansions of typical temperate species originate from their Balkan lineages during transitions from cold to warm periods, the Balkan lineages usually represent the endpoints of colonisations for cold-adapted mountain species, reaching the region from the eastern Alps and the southern Carpathians, respectively^44^. Consequently, although *E. aethiops* has to be considered a temperate forest species, it behaves like a mountain taxon in this context. Furthermore, in contrast to boreal taxa, which have been expanding into this region during ice ages, the expansion of *E. aethiops* most likely took place during warmer periods, in which forests were spreading over large parts of this region, thus facilitating the species’ dispersal.

According to our genetic data, the colonisation of the before mentioned western Balkan Peninsula from the eastern Alps and the eastern Balkan Peninsula from the southern Carpathians happened within similar time horizons. However, the almost complete lack of differentiation between the eastern Balkans and the southwestern Carpathians (population Baile Herculane) supports the theory of an expansion via the Iron Gate (i.e. crossing of the Danube through the south-western Carpathians) to the Balkan Peninsula, not before the Eem interglacial. During the same time frame as this southern expansion, quick expansions out of the geographically strongly restricted Riss glacial refugia in the Carpathian region must have taken place. These expansions (that must have been associated with the complete reforestation of this region) apparently led to an area-wide distribution of *E. aethiops,* which is in line with the results of our Bayesian Skyline analysis. This hypothesis is also supported by the genetic patterns at the nuclear level, for which no detectable genetic differentiation in the southern Carpathian region exists. The mitochondrial level, however, might largely reflect the Riss glacial refugia, an apparent contradiction, which might be explained by different dispersal potentials of the sexes^20,22^, but which needs to be investigated in more depth in future work.

The expansion within the Carpathian region during the Eem interglacial must have covered the entire arc up to the Tatras. However, both nuclear and mitochondrial data show that both the Tatras and the eastern Carpathians were populated by *E. aetiops* not only from the southern Carpathians, but also from a centre of dispersal in the eastern Alps. Such a mixture of Alpine and Carpathian elements of certain taxa in the Tatras has already been demonstrated several times^10,11^. However, the existence of numerous endemic haplotypes and allozyme alleles in the Tatras also suggests that *E. aethiops* not only colonised this region during the Eem interglacial, but was also able to survive the relatively mild Würm glacial there. A similar situation has been indicated for the eastern Carpathians, but the available data is not as robust as in the case of the Tatras. Our data thus show that not only individual trees must have been present on the southern fringe of the Tatras (and presumably also in the eastern Carpathians) during the last glacial period^8,14,17,41,42,45^, but that there were local refugia in which temperate forests have survived permanently.

If one considers the species patchy distribution or lack at the Mediterranean peninsulas and the insufficient coverage of the Siberian Taiga, it becomes evident that *E. aethiops'* occurrence is also strongly influenced by its own biogeographic history. Therefore, the species cannot be considered as an indicator species of the entire western Palaearctic forest ecosystem, but within its range it can enrich the knowledge about the history and dynamics of deciduous forest structures. Thus, due to its mobility and sensitive response to environmental changes, it reacts reliably to changes in its ecosystem. Therefore, its recent distribution and genetic structure carries information not only on its own evolutionary history, but also on that of its associated ecosystem.

## Material and methods

### Study species

The Scotch Argus *Erebia aethiops* is a univoltine species with overwintering larvae. Eggs are laid on a wide spectrum of different grasses (Poaceae, e.g. *Molinia caerulea*, *Brachypodium pinnatum*, *Festuca* ssp., *Dactylis glomerata*, *Poa* ssp.), but also, less commonly, on sedges (Cyperaceae, e.g. *Luzula nivea*, *Carex ferruginea*, *Carex sempervirens*)^46^. The caterpillars pupate end of June, resulting in a flight season starting in July and ending at the beginning of September, with the main activity in late July and August.

### Sampling design

**Figure 5:**
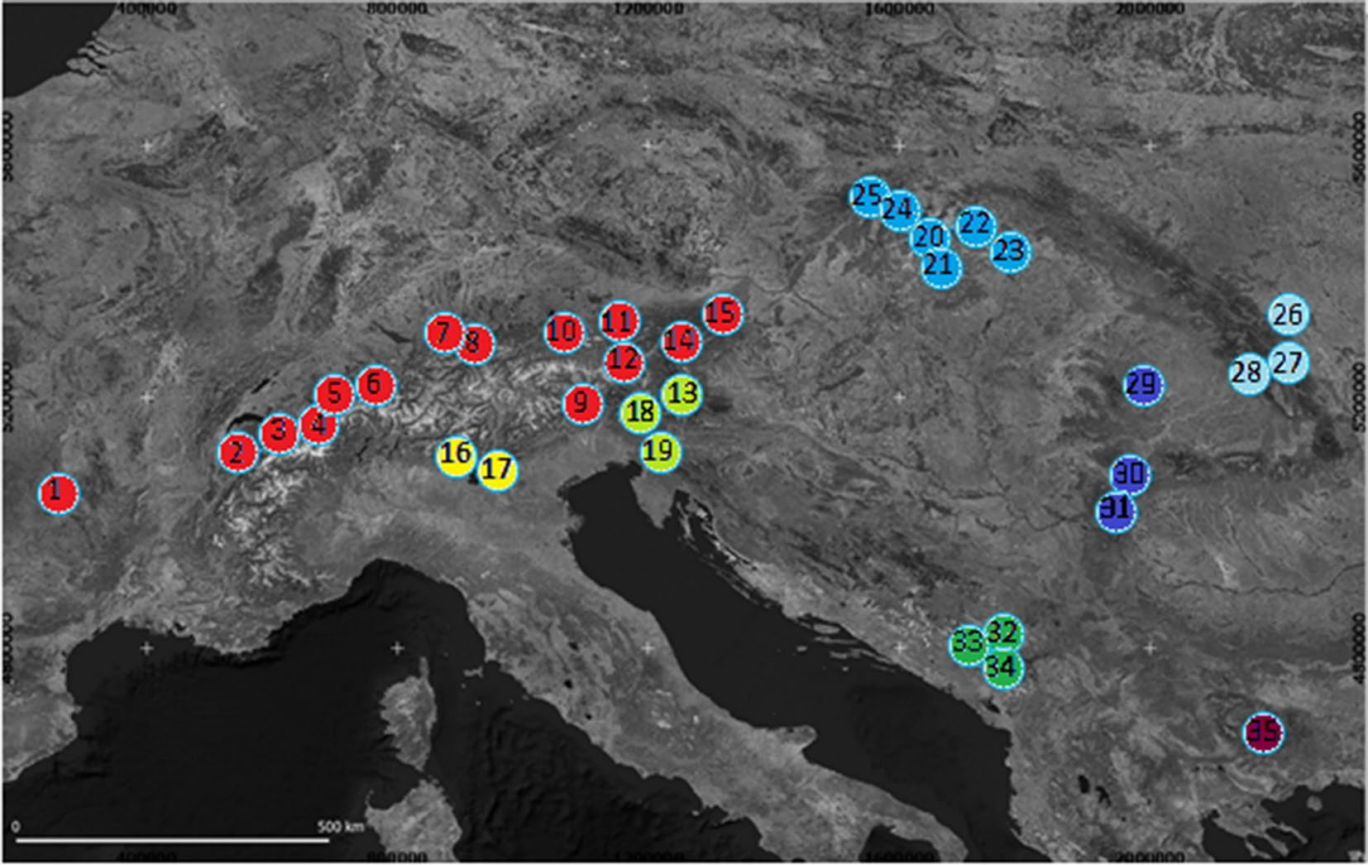
Geographic location of the sampled population of *E. aethiops*. Colors indicate the different genetic groups detected by genetic analyses. The map was created with Qgis v.3.10.10^72^ (Available online: http://qgis.osgeo.org).

859 *E. aethiops* individuals representing 35 populations (8–41 specimens, mean: 30.8 per population) were sampled from 2002 to 2014 from the Massif Central in France to the Romanian Carpathians and Bulgarian mountain systems (Fig. 5, supplementary table S14). The butterflies were captured with a hand net in the field and frozen in liquid nitrogen. Specimens were subsequently stored in liquid nitrogen or in a refrigerator at − 80 °C until analyses. We added 48 sequences from BOLD and Genbank (accession numbers in Table S15 and distribution map in Fig. S16 of the supplementary) to get genetic data from parts of the range not covered by our own sampling. Since these just represented COI sequences, partly fragmentary (429 bp), only a simple comparison was possible and not a complete analysis. The sample areas did not require permissions, except for Austria and Slovakia, whose environmental offices had issued these permits.

### Genetic analyses

#### DNA sequencing

Total genomic DNA was extracted from one leg using the E.Z.N.A. Tissue DNA Kit (Omega Bio-Tek, Norcross, USA) following the manufacturer’s protocol.

Variation of two mitochondrial genes was assessed for a subset of 133 individuals representing 28 populations covering all eight regions (see supplementary S14). We sequenced the barcoding region of the cytochrome c oxidase subunit I (COI, 658 bp), which provides reliable resolution at the intraspecific level as shown in several previous studies on *Erebia*^47,48^, as well as the NADH dehydrogenase subunit I (NDI, 554 bp). COI was amplified with the primer pair LEP-F1 (5′-ATTCAACCAATCATAAAGATATTGG-3′) and LEP-R1 (5′-TAAACTTCTGGATGTCCAAAAAATCA-3′)^49^ applying the following PCR protocol: 95 °C for 5 min, followed by 38 cycles at 95 °C for 30 s, 49 °C for 90 s, 72 °C for 60 s and terminated with a final extension step at 68 °C for 30 min. NDI was amplified using the primer pair FAW-NDI (5′-TTCAAACCGGTGTAAGCCAGG-3′) and FAW-16S (5′-TAGAATTAGAAGATCAACCAGC-3′)^50^ and the following PCR protocol: 95 °C for 5 min, 33 cycles at 95 °C for 30 s, 56 °C for 90 s, 72 °C for 60 s and terminated at 68 °C for 30 min. PCR products were visualized on 1.4% agarose gels, stained with GelRed (Biotium, Fremton, USA). Amplified products were purified with a mix of FastAP and Exonuclease I (Thermo Scientific, Dreieich, Germany) and sent to Macrogen Europe (Amsterdam, Netherlands) for sequencing in both directions with the same two primers as were used for PCR.

#### Allozymes

836 individuals from 27 populations (see supplementary S14) were analysed by allozyme electrophoresis. We analyzed a total of 20 allozyme loci (i.e. 6PGDH, IDH1, IDH2, GPDH, G6PDH, GAPDH, FUM, MDH1, MDH2, ME, GOT1, GOT2, PEP LGG; MPI, PK1, PK2, PK3, PGM, PGI1, PGI2) following the standard protocol by^51^. Running conditions were applied as in Schmitt and Seitz ^32^.

### Statistical analyses

#### Mitochondrial DNA

Sequences were assembled with Geneious v. 10.2.3^52^ and aligned using ClustalW^53^ implemented in BioEdit v. 7.2.6.1^54^. Sequences of both genes were concatenated and checked for stop-codons with Geneious. Genbank accessions are given in Table S17 of the supplementary. The frequency of haplotypes, haplotype diversity (h), number of segregating sites (S), nucleotide diversity per gene (p_*i*_), and the average number of nucleotide differences (k) were calculated with DNAsp v. 6^55^. The concatenated file was used to construct a Minimum Spanning haplotype network^56^ with PopArt v. 1.7^57^.

A Bayesian tree was reconstructed based on the mitochondrial data with BEAST v. 2.5^58^. Published data of *Pararge aegeria* (GenBank accessions in Table S17 of the supplementary) and own mtDNA sequences of *Erebia pronoe* were used as outgroups. Partitions and substitution models were estimated with Partitionfinder v. 2.1.1^59^ based on the lowest Akaike Information Criterion (AIC). The HKY model with empirical base frequencies and a Gamma distribution with a category count of 4 was selected. We ran several analyses to select the best fitting tree model. The coalescent constant population model performed best and hence was chosen for the final analysis. The molecular clock was calibrated with a substitution rate of 0.0177^60^. We ran the analyses with 40 million generations sampling every 4,000 iterations. After checking the MCMC chain for convergence in Tracer v. 1.7.1^61^, a burn-in of 10% was applied. Three individual runs were performed and combined using LogCombiner v. 1.8.4^62^**.** TreeAnnotator v. 2.5^62^ was used to generate a summary tree with common ancestor heights. Figtree v. 1.4.4^61^ was used for visualisation.

The same protocol was followed to obtain an input and consensus tree for a reconstruction of ancestral area analysis with RASP v. 4.2^63^. This analysis was based on the extended COI data set of *E. aethiops* and further COI sequences of *E. neriene*, *E. niphonica* and *E. alcema* obtained from Genbank and Boldsystems. The additional accession numbers are given in the RASP tree. The individual sequences were assigned occurrences coded as consecutive letters (A: Sakalin; B: Hoshu; C: zone of sympatry between *E. aethiops* and *E. neriene* in the Altai Mountains; D: Mongolia; E: China; F: Europe; G: western Altai; H: Turkey; J: Ural; I: Caucasus). Based on the results of the BioGeoBears model test, an S-DEC analysis was performed with the maxarea = 6 setting.

To model the demographic history of the populations, a Bayesian Skyline plot was generated with BEAST. Bayesian skyline analyses were run with the coalescent tree prior, in a single run with 40 million generations, log parameters were sampled every 4,000 iterations. The effective sample size and female population size was evaluated using Tracer v. 1.7.1. We used the substitution rate estimated by Papadopoulou et al.^60^.

#### Allozymes

Alleles were labeled according to their relative mobility, starting with “1” for the slowest. Their frequency, the total number of alleles per locus (A) and further genetic diversity parameters (i.e. expected and observed heterozygosity (H_e_; H_o_), total number of polymorphic loci (P_tot_) and the percentage of polymorphic alleles per locus with the most common allele not exceeding 95% (P95)) were estimated with G-Stat v. 3^64^. Tests on Hardy–Weinberg equilibrium (HWE) and linkage disequilibrium (LD) were performed with Arlequin v 3.5^65^. A sequential Bonferroni correction^66^ was applied. A non-hierarchical and a hierarchical genetic variance analysis (AMOVA) were performed with Arlequin to attribute the genetic variation to the following levels: among populations, among individuals within populations, and within individuals. Additionally, pairwise F_ST_ values were estimated with Arlequin. A Neighbor Joining (NJ) phenogram^67^ based on Nei’s genetic distance^26^ and 1,000 bootstrap replicates was generated with Phylip v. 3.67^68^. Structure v. 2.3.4^69^ was used to infer the number of genetic clusters without a priori definition of populations. To ensure a better delimitation of the individual groups, which were suggested by Structure, each population was examined for a change in the main allele. We tested for genetic clusters (K) from two to 27 (i.e. the number of populations analyzed for allozymes). Ten replicates were carried out for each K, with a burn-in of 100,000 and 500,000 MCMC replicates performed thereafter. Due to the un-reliability of logarithmic probabilities, an ad hoc method was used and the ∆K value, based on the change of the logarithmic probability of successive K values, was determined^70^. The high ∆K value for K = 2 was ignored a suggested by Hausdorf and Hennig^27^. Additionally, a Geneland^71^ analysis was conducted with ten runs, each of 250,000 generations, in an uncorrelated frequency model with the number of classes treated as unknown and variable along the MCMC chain.

## Supplementary information

Supplementary information.

## Data Availability

The datasets used and/or analysed during the current study are available from the corresponding author on reasonable request. All data generated or analysed during this study are included in this published article [and its supplementary information files].
