# Supplementary material for: Reconstruction of forest dynamics in the Western Palaearctic based on phylogeographic analysis of the ringlet butterfly Erebia aethiops
Source: Sci Rep. 2021 Jan 8;11:201. doi: 10.1038/s41598-020-79376-x (PMC7794548; doi:10.1038/s41598-020-79376-x)
Supplement: Supplementary file 1 — Supplementary information. [file 41598_2020_79376_MOESM1_ESM.doc]

**Reconstruction of forest dynamics in the Western Palaearctic based on phylogeographic analysis of the ringlet butterfly *Erebia aethiops***

Running head: Butterflies and forest dynamics

Martin Wendt^a^*, Martin Husemann^b^, Katja Kramp^c^, Thomas Schmitt^a,d^

*^a^Senckenberg Deutsches Entomologisches Institut, Systematik und Biogeographie, Eberswalder Str. 90, 15374 Müncheberg, Germany*

*^b^Universität Hamburg, Centrum für Naturkunde, Martin-Luther-King-Platz 3, 20146 Hamburg, Germany*

*^c^Leibniz-Zentrum für Agrarlandschaftsforschung (ZALF) e.V., PB 2: „Landnutzung und Governance“, AG: Biotische Interaktionen zwischen Wald- und Agrarflächen, Eberswalder Str. 84, 15374 Müncheberg, Germany*

*^d^ Zoology, Institute of Biology, Faculty of Natural Sciences I, Martin Luther University Halle-Wittenberg, 06099 Halle (Saale), Germany*

- Corresponding author. Tel.: +49 (0) 33432 73698-3755. E-mail address: [**martin.wendt@senckenberg.de**](mailto:martin.wendt@senckenberg.de) (M. Wendt)

**Supplementary data**

**
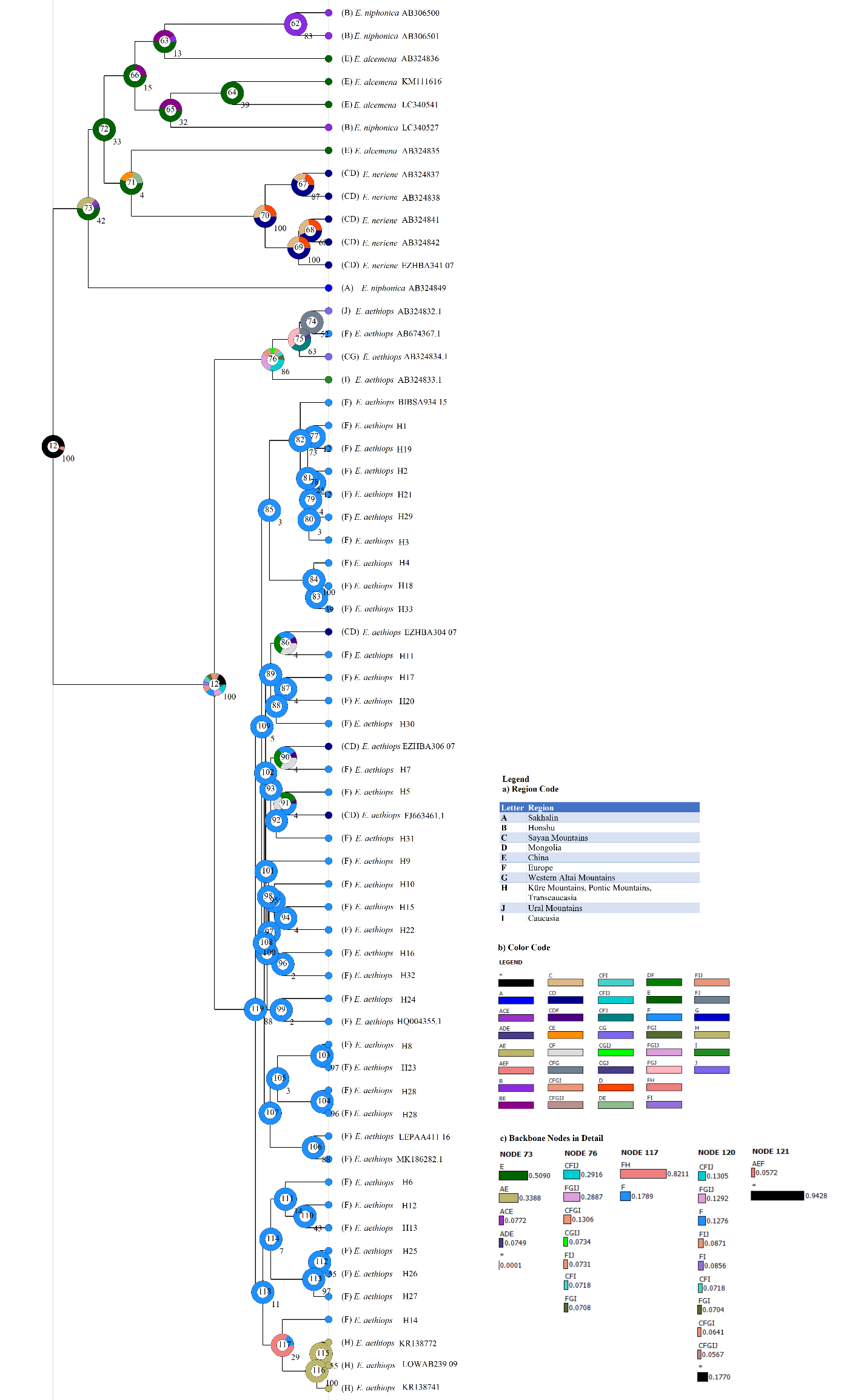
**

S1: Graphical output from Dispersal-Extinction-Cladogensis (DEC) analysis (exported from RASP). Graphical results of ancestral distributions at each node of the *aethiops* group obtained by DEC analysis. Pie charts at each node show probabilities of alternative ancestral ranges with the posterior probability for the node next to it. a) Region codes; b) Color key to possible ancestral ranges at different nodes; black with an asterisk represents other ancestral ranges; c) backbone nodes in detail. Tip labels have the area codes and the accessions number or rather the haplotype number.

| **Model** | **LnL** | **parameters** | **AICc** |
| --- | --- | --- | --- |
| DEC | -110.8 | 2 | 225.9 |
| DEC+J | -106.2 | 3 | 218.8 |
| DIVALIKE | -103.9 | 2 | 212 |
| DIVALIKE+J | -101.6 | 3 | 209.5 |
| BAYAREALIKE | -108.1 | 2 | 220.5 |
| BAYAREALIKE+J | -93.14 | 3 | 192.7 |

S2: Model test by RASP v. 4.2 with log of the Likelihood, number of parameters and corrected Akaike Information Criterion.


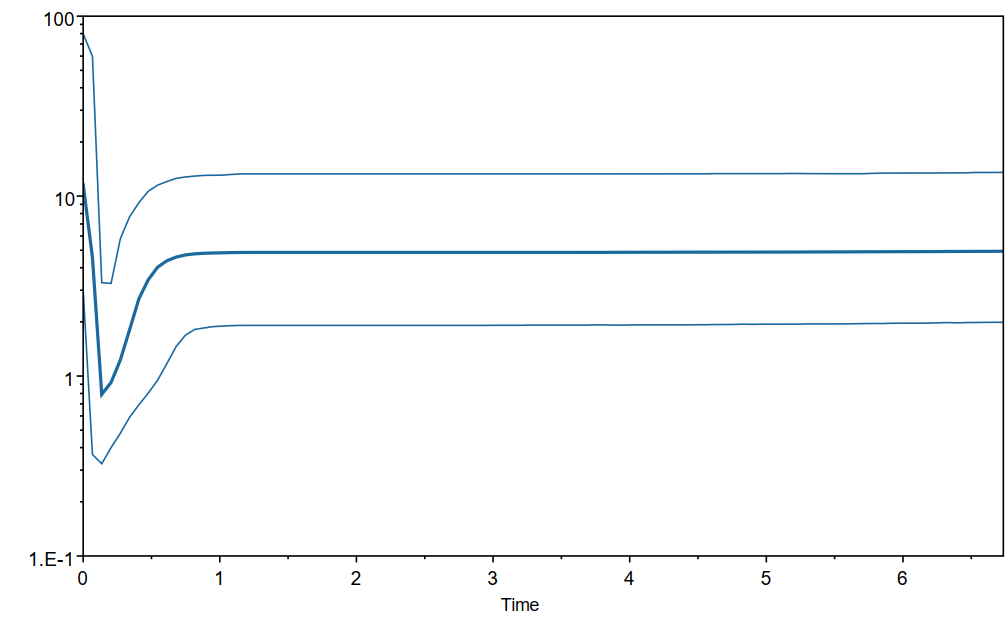


S3: Bayesian Skyline Plot of *E. aethiops* based on mtDNA data.

| Country | Location | He | Ho | A | Ptot | P95 | N |
| --- | --- | --- | --- | --- | --- | --- | --- |
| F | Artout | 10,1 | 10,4 | (1,39) | (33,3) | (33,3) | 8,0 |
| F | Col des Aravis | 4,6 | 4,8 | (1,28) | (22,2) | 16,7 | 14,0 |
| CH-VS | Ausserberg | 6,0 | 5,4 | 1,44 | 38,9 | 16,7 | 42,0 |
| CH-BE | Grindelwald | 6,8 | 5,3 | (1,33) | (22,2) | 16,7 | 18,8 |
| CH-UR | Klausenpass | 6,2 | 6,5 | 1,50 | 38,9 | 16,7 | 36,0 |
| A-NT | Fernpass | 9,3 | 7,2 | 1,67 | 61,1 | 33,3 | 38,9 |
| A-NT | Holzleithner Sattel | 6,4 | 5,6 | 1,83 | 72,2 | 33,3 | 39,5 |
| I | Plöckenpass | 5,8 | 4,9 | 1,44 | 33,3 | 11,1 | 37,7 |
| A-SB | Sonnenstein | 8,3 | 7,8 | 1,94 | 66,7 | 22,2 | 39,9 |
| A-ST | Loser | 8,4 | 9,0 | (1,40) | (44,4) | (44,4) | 8,0 |
| A-SB | Seetal | 5,3 | 5,3 | 1,44 | 27,8 | 11,1 | 36,9 |
| SLO | Medvodje | 6,2 | 5,3 | 1,72 | 50,0 | 27,8 | 37,5 |
| A-ST | Kaisertal | 7,7 | 6,6 | 1,61 | 55,6 | 33,3 | 39,3 |
| A-ST | Seebergsattel | 8,1 | 7,1 | 1,83 | 61,1 | 27,8 | 37,6 |
| I | Spiazzi | 4,8 | 4,6 | 1,56 | 33,3 | 22,2 | 26,0 |
| I | Campolaro | 8,2 | 7,6 | 2,00 | 55,6 | 27,8 | 38,0 |
| SK | Tokariny | 6,8 | 7,8 | 1,67 | 50,0 | 11,1 | 36,9 |
| SK | Pusté pole | 11,2 | 9,7 | 2,06 | 66,7 | 38,9 | 36,0 |
| H | Szelcepuszta | 7,8 | 8,2 | (1,39) | 27,8 | 27,8 | 21,1 |
| H | Haragistya | 10,9 | 12 | 1,72 | 61,1 | 38,9 | 27,8 |
| SK | Kojšovska holá | 6,9 | 6,6 | 1,72 | 50,0 | 22,2 | 37,7 |
| H | Gyertyánkút | 10,0 | 9,4 | (1,39) | (33,3) | 27,8 | 14,9 |
| RO | Baile Herculane | 7,0 | 7,1 | (1,33) | (22,2) | (22,2) | 7,9 |
| RO | Cheile Buţii | 10,5 | 10,0 | 1,89 | 61,1 | 33,3 | 37,9 |
| RO | Cheile Runcului | 11,2 | 11,7 | 1,78 | 55,6 | 27,8 | 39,8 |
| SLO | Nanos | 9,3 | 9,4 | 1,78 | 50,0 | 27,8 | 40,7 |
| BG | Trigrad | 14,1 | 14,0 | 1,61 | 50,0 | 27,8 | 35,7 |
|  | MEAN | 8,1 | 7,8 | 1,62 | 46,1 | 26,0 |  |
|  | SD | 2,3 | 2,5 | 0,22 | 15,1 | 8,8 |  |
| F | *C. oedippus* | 25,3 | 20,1 | 2,88 | 87,5 | 62,5 | 95,5 |

S4: Allozyme diversity patterns of *E. aethiops*. The following values are given: mean number of alleles A, percentage of the expected and observed heterozygosity He and Ho, percentage of all polymorphic loci Ptot and of loci with the most common allele not exceeding 95 % P95. Values based on an insufficient number of individuals are given in parenthesis and are excluded from the calculation of means.

| Population/ Linkagepairs | Medvodje | Nanos | Szelcepuszta | Sonnenstein | Kaisertal | Grindelwald |
| --- | --- | --- | --- | --- | --- | --- |
| 1 | GOT1-GOT2 | 6PGDH-IDH1 | 6PGDH-GOT2 | 6PGDH-PEP | 6PGDH-GOT1 | IDH2-PGM |
| 2 | GOT1-ME | 6PDGH-IDH2 | 6PGDH-PGM |  |  |  |
| 3 | GOT1-PGI | IDH1-IDH2 | GOT2-PGM |  |  |  |
| 4 | GOT2-ME |  |  |  |  |  |
| 5 | GOT2-PGI |  |  |  |  |  |
| 6 | ME-PGI |  |  |  |  |  |

S5 Linkage pairs of alleles by populations of *E. aethiops.*

|  | Central Alps | Eastern Balkan | Southern Alps | Southern Carpathians |
| --- | --- | --- | --- | --- |
| Eastern Balkan | 0,079  ±0,012 |  |  |  |
| Southern Alps | 0,153  ±0,018 | 0,169  ±0,008 |  |  |
| Southern Carpathians | 0,059  ±0,018 | 0,046  ±0,004 | 0,121  ±0,008 |  |
| Western Balkan | 0,056  ±0,016 | 0,059  ±0,0 | 0,209  ±0,014 | 0,108  ±0,010 |

S6: Mean genetic distance of the four main geo. groups of *E. aethiops* based on the allozyme data.


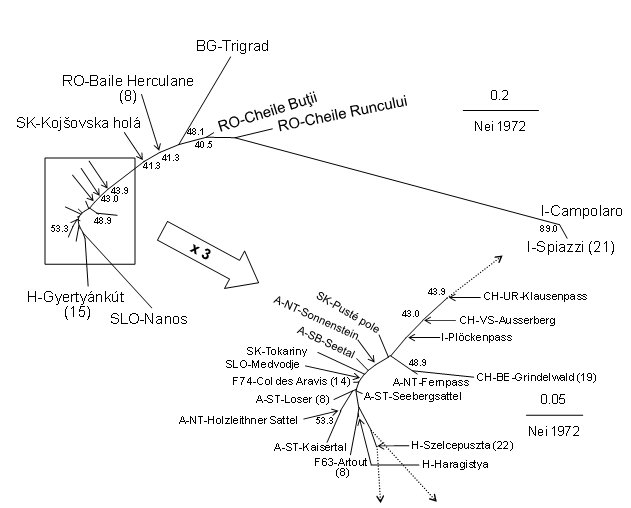


S7: Neighbor Joining phenogram based on the genetic distance (Nei 1972) of 27 populations of *E. aethiops*. Bootstrap values > 40 % are given at the branches. The box was magnified three times.

| K | LN(PR) | ±SD | ΔK |
| --- | --- | --- | --- |
| 1 | -6712.8500 | 0.0527 | NA |
| 2 | -6079.6000 | 2.7673 | 180.412529 |
| 3 | -5945.6000 | 23.6026 | 8.210523 |
| 4 | -5617.8100 | 28.2878 | 8.214135 |
| 5 | -5522.3800 | 4.3645 | 25.423579 |
| 6 | -5537.9100 | 22.3992 | 0.609397 |
| 7 | -5539.7900 | 12.1479 | 2.474496 |
| 8 | -5511.6100 | 14.1166 | 0.570250 |
| 9 | -5491.4800 | 28.7081 | 2.599966 |
| 10 | -5545.9900 | 36.9710 | 1.188769 |
| 11 | -5644.4500 | 36.9469 | 0.900481 |
| 12 | -5709.6400 | 27.1023 | 1.718303 |
| 13 | -5821.4000 | 124.6410 | 0.816264 |
| 14 | -6034.9000 | 218.3150 | 0.886792 |
| 15 | -6054.8000 | 202.7125 | 0.849134 |
| 16 | -6246.8300 | 214.2018 | 0.991728 |
| 17 | -6226.4300 | 192.6670 | 0.634255 |
| 18 | -6328.2300 | 189.4610 | 0.236566 |
| 19 | -6474.8500 | 261.2760 | NA |

S8: Structure K-values. Estimates of cluster number (K) from STRUCTURE analyses using allozyme polymorphisms for (a) all species and populations analysed, calculated for K= 1–27. Ln(Pr) is the mean log-likelihood probability calculated by the program STRUCTURE. SD is the standard deviation calculated from ten independent runs. The ad hoc statistic ΔK is not applicable for K= 1 and the highest K value, and not proper for K= 2 (Hausdorf and Hennig 2010).

**
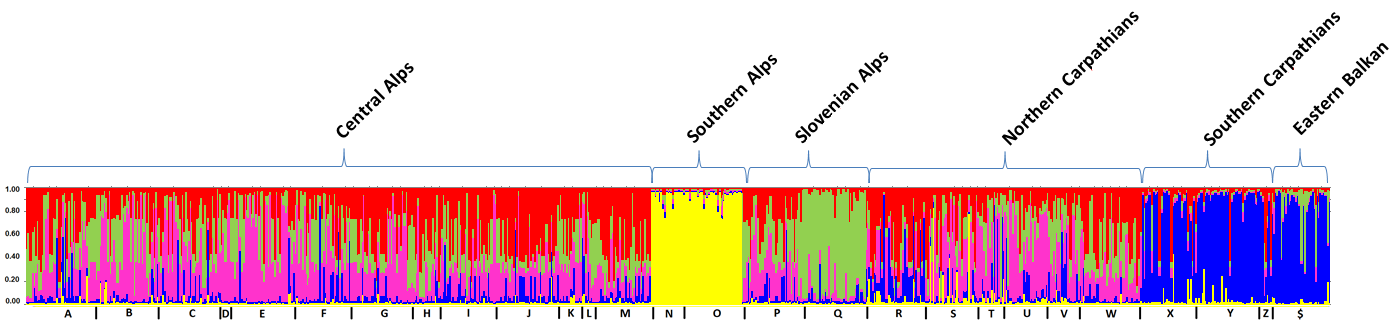
**

S9: Bayesian structure analysis of the *E. aethiops* populations using STRUCTURE software (Pritchard et al., 2000). The analysis was performed for five given groups (K = 5).

A:Fernpass,B:Holzleitner Sattel, C:Sonnenstein, D:Loser, E:Kaisertal, F:Seebergsattel, G:Seetal, H:Grindelwald, I:Klausenpass, J:Ausserberg, K:Col des Aravis, L:Artout, M:Ploeckenpass,N:Spiazzi, O:Campolaro, P:Medvodje, Q:Nanos, R: Kojšovska holá, S: Pusté pole, T: Gyertyánkút, U: Haragistya, V: Szelcepuszta, W: Tokariny, X: Cheile Buţii, Y: Cheile Runcului, Z: Baile Herculane, $:Trigrad


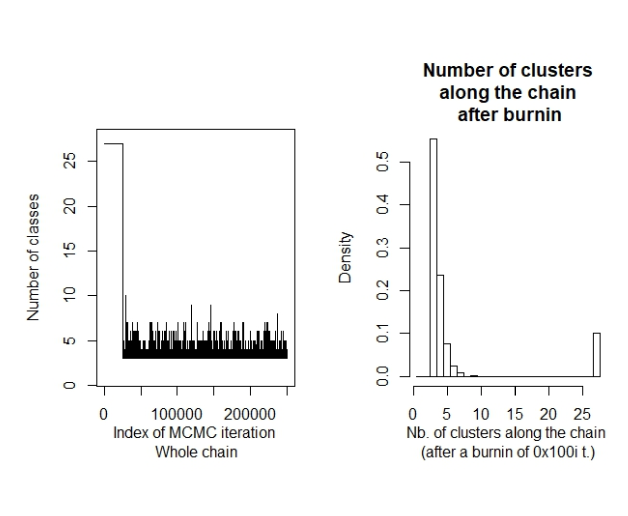


S10: Number of populations along the MCMC run by Geneland.


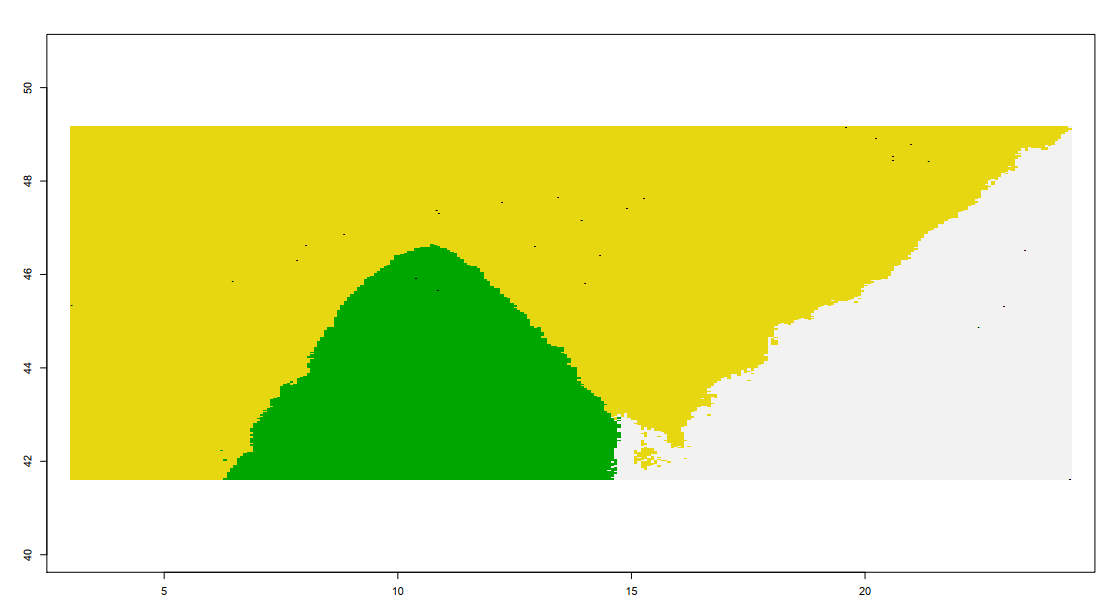


S11: Map of population membership by Geneland based on the allozyme data of *E. aethiops*

|  | Central Alps | Eastern Balkan | Central Italian Alps | Southern Carpathians |
| --- | --- | --- | --- | --- |
| Eastern Balkan | 0,284  ± 0,043 |  |  |  |
| Southern Alps | | 0,567 | | --- | | ± 0,046 | | | 0,498 | | --- | | ± 0,013 | |  |  |
| Southern Carpathians | | 0,276 | | --- | | ± 0,080 | | | 0,164 | | --- | | ± 0,020 | | | 0,467 | | --- | | ± 0,034 | |  |
| Western Balkan | | 0,243 | | --- | | ± 0,053 | | 0,224  ±0,0 | | 0,623 | | --- | | ± 0,004 | | | 0,416 | | --- | | ± 0,017 | |

S12: Pairwise F_ST_ values of the four main geo. groups of *E. aethiops* based on the allozyme data

| Location | Switch in the main allele | Endemic alleles |
| --- | --- | --- |
| Fernpass |  |  |
| HolzleithnerSattel |  |  |
| Sonnenstein |  | PGM-1 |
| Loser |  |  |
| Kaisertal |  |  |
| Seebergsattel |  | IDH2-1 |
| Seetal |  |  |
| Grindelwald |  | ME-1 |
| Klausenpass |  |  |
| Ausserberg | PGM-3(4) |  |
| Col des Aravis |  |  |
| Artout |  | GOT-6 |
| Plöckenpass |  |  |
| Medvodje |  |  |
| Haragistya |  |  |
| Szelcepuszta |  |  |
| Gyertyánkút |  |  |
| Tokariny |  |  |
| Pusté pole |  | GOT-4, MDH1-1, MDH1-3 |
| Spiazzi | PEP-3(2), PGM-3(4) | G6PDH-1, GOT-5 |
| Campolaro | GOT2-1(3), PEP-3(2), PGM-3(4) | FUM-1, GOT-2 |
| Kojšovskaholá | PGM-3(4) |  |
| CheileBuţii | PGM-3(4) |  |
| CheileRuncului | IDH1-5(2), PGM-3(4) | IDH1-3, MDH1-4 |
| BaileHerculane | PGM-3(4) |  |
| Nanos |  |  |
| Trigrad | IDH1-5(2), PGI-3(5), PGM-3(4) | IDH2-5 |

S13: Private allele and changes of the main allele by populations of *E. aethiops*

| **No.** |  | **Location** | **Hight [m NN]** | **Coordinates** |
| --- | --- | --- | --- | --- |
|  |  | Artout | 1250 | 45°20'16''N 03°01'25''E |
|  |  | Col des Aravis | 1700 | 45°51'54''N 06°27'22''E |
|  |  | Val-d´Ílliez | 890 | 46°07'03''N 07°01'19''E |
|  |  | Ausserberg | 1200 | 46°19'10''N 07°51'33''E |
|  |  | Grindelwald | 1800 | 46°38'43''N 08°01'44''E |
|  |  | Klausenpass | 1800 | 46°52'21''N 08°52'18''E |
|  |  | Fernpass | 1250 | 47°21'53''N 10°50'01''E |
|  |  | HolzleithnerSattel | 1100 | 47°18'24''N 10°53'13''E |
|  |  | Plöckenpass | 1450 | 46°36'10''N 12°56'13''E |
|  |  | Sonnenstein | 1200 | 47°32'58''N 12°15'55''E |
|  |  | Loser | 1550 | 47°39'29''N 13°26'46''E |
|  |  | Seetal | 1300 | 47°09'11''N 13°56'16''E |
|  |  | Medvodje | 1550 | 46°25'25''N 14°19'02''E |
|  |  | Kaisertal | 1050 | 47°25'08''N 14°54'31''E |
|  |  | Seebergsattel | 1300 | 47°37'44'‘N 15°16'44''E |
|  |  | Spiazzi | 900 | 45°39'26''N 10°51'18''E |
|  |  | Campolaro | 1550 | 45°54'37''N 10°23'04''E |
|  |  | Sija | 500 | 46°11'41''N 13°50'01''E |
|  |  | Nanos | 800 | 45°48'22''N 14°00'51''E |
|  |  | Szelcepuszta | 500 | 48°31'35''N 20°35'02''E |
|  |  | Haragistya | 550 | 48°26'30''N 20°32'18''E |
|  |  | Kojšovskaholá | 1100 | 48°46'35''N 20°59'11''E |
|  |  | Gyertyánkút | 700 | 48°26'30''N 21°21'52''E |
|  |  | Pusté pole | 950 | 48°53'10''N 20°14'21''E |
|  |  | Tokariny | 800 | 49°09'45''N 19°37'49''E |
|  |  | Pascani | 460 | 47°06'23''N 26°24'38''E |
|  |  | Covacipeter | 1360 | 46°40'40''N 25°43'13''E |
|  |  | Gheorgheni | 980 | 46°34'45''N 25°33'46''E |
|  |  | CheileRuncului | 600 | 46°30'48''N 23°26'18''E |
|  |  | CheileBuţii | 950 | 45°18'00''N 22°58'29''E |
|  |  | BaileHerculane | 350 | 44°51'57''N 22°25'02''E |
|  |  | Tresnjevik | 1580 | 42°44'19''N 19°41'01''E |
|  |  | Ropojantal | 1400 | 42°29'13''N 19°48'27''E |
|  |  | Valbona | 1350 | 42°30'01''N 19°56'50''E |
|  |  | Trigrad | 1350 | 41°36'32''N 24°23'45''E |

S14: Geographical sample location of all *E. aethiops* samples including geographic coordinates and altitude.

| **Accession number** | **Species/Color code of *E. aethiops*** | **Database** | **Location** |
| --- | --- | --- | --- |
| MK186285.1 | red | Genbank | Graubunden Piz Beverin Switzerland |
| MK186282.1 | orange | Genbank | Bern Neuveville Switzerland |
| MK186281.1 | yellow | Genbank | Locarno Switzerland |
| KT782504.1 | orange | Genbank | Riga Latvia |
| HQ563606.1 | orange | Genbank | Bavaria Schmidmühlen Germany |
| HQ004360.1 | light blue | Genbank | Suceava pas Pascanu Romania |
| HQ004359.1 | light blue | Genbank | Suceava ValeaBistritei Romania |
| HQ004358.1 | light blue | Genbank | Brasov Racos Romania |
| HQ004357.1 | light blue | Genbank | Suceava Sunatori Romania |
| HQ004355.1 | light blue | Genbank | Brasov cabana Babarunca Romania |
| HM391826.1 | red | Genbank | Bavaria Wimbachschloss Germany |
| FJ663461.1 | brown | Genbank | East Kazakhstan |
| KX040921.1 | red | Genbank | Bavaria Oytal Germany |
| AB324832.1 | brown | Genbank | Middle Urals Serga river Russia |
| AB324834.1 | brown | Genbank | Alan Range Sayan Russia |
| AB324833.1 | brown | Genbank | Caucasus Teberda Russia |
| KR138772 | pink | Genbank | Achalkalaki Georgia |
| KR138741 | pink | Genbank | Ersizlerdere Turkey |
| MN138605 | red | Genbank | Bavaria Oberstdorf Germany |
| BIBSA1199-15 | gold | Bold | Vipiteno Sant Antonio Italy |
| BIBSA125-15 | gold | Bold | Liguria San Martino Italy |
| BIBSA201-15 | gold | Bold | Alpedella Gianna Italy |
| BIBSA934-15 | gold | Bold | Rifugio Monte Mongioie Italy |
| EULEP2778-15 | orange | Bold | Isle of Arran Scotland |
| EULEP593-15 | red | Bold | Isere France |
| EULEP617-15 | orange | Bold | Thüringen Altremda Germany |
| EULEP980-15 | red | Bold | Ginzlingen Austria |
| EZHBA304-07 | brown | Bold | Iskitim Russia |
| EZHBA306-07 | brown | Bold | Iskitim Russia |
| GBLAB766-13 | orange | Bold | Bavaria Fischerhäuser Isarauen Germany |
| GWORR684-10 | red | Bold | Bavaria Jachenau Germany |
| LEATC177-13 | gold | Bold | Margreid Italy |
| LEPAA411-16 | orange | Bold | Waldenburg Switzerland |
| LEPAA839-16 | red | Bold | Uznach Switzerland |
| LOWAB239-09 | pink | Bold | Ispir Turkey |
| OXB258-15 | orange | Bold | Kirkby Stephen Smardale Great Britain |
| OXB259-15 | orange | Bold | Kirkby Stephen Smardale Great Britain |
| OXB260-15 | orange | Bold | Kirkby Stephen Smardale Great Britain |
| OXB603-15 | yellow | Bold | Madonna di Campiglio Italy |
| OXB724-15 | gold | Bold | Col de Visentin Italy |
| PHLAI521-13 | red | Bold | Greit Austria |
| WMB1643-13 | red | Bold | Isere France |
| WMB1867-13 | red | Bold | Col du Noyer France |
| WMB3680-14 | red | Bold | Piste Sky Laye France |
| WMB5027-14 | gold | Bold | Monte Calvaria France |
| WMB837-13 | gold | Bold | Mompantero Vecchio Italy |
| AB324849 | *E. niphonica* | Genbank | Sakhalin Reg. Russia |
| LC340527 | *E. neriene niphonica* | Genbank | Akaishi Mts. Japan |
| AB306501 | *E. niphonica* | Genbank | Ishikawa Japan |
| AB306500 | *E. niphonica* | Genbank | Nagano Japan |
| EZHBA341-07 | *E. neriene* | Bold | Todzha interm Russia |
| AB324842 | *E. neriene* | Genbank | Hangay Mts. Mongolia |
| AB324841 | *E. neriene* | Genbank | Terelj Mongolia |
| AB324838 | *E. neriene* | Genbank | Khabarovsk Russia |
| AB324837 | *E. neriene* | Genbank | Khabarovsk Russia |
| KM111616 | *E. alcmena* | Genbank |  |
| AB324835 | *E. alcmena* | Genbank | Shaanxi China |
| LC340541 | *E. alcmena* | Genbank | Datong-Shan China |
| AB324836 | *E. alcmena* | Genbank | Qinghai China |
|  |  |  |  |

S15: Bold and Genebank accession numbers of COI sequences fragment (429bp) of *E. aethiops with colour code,* *E. neriene, E. niphonica und E. alcmena.*


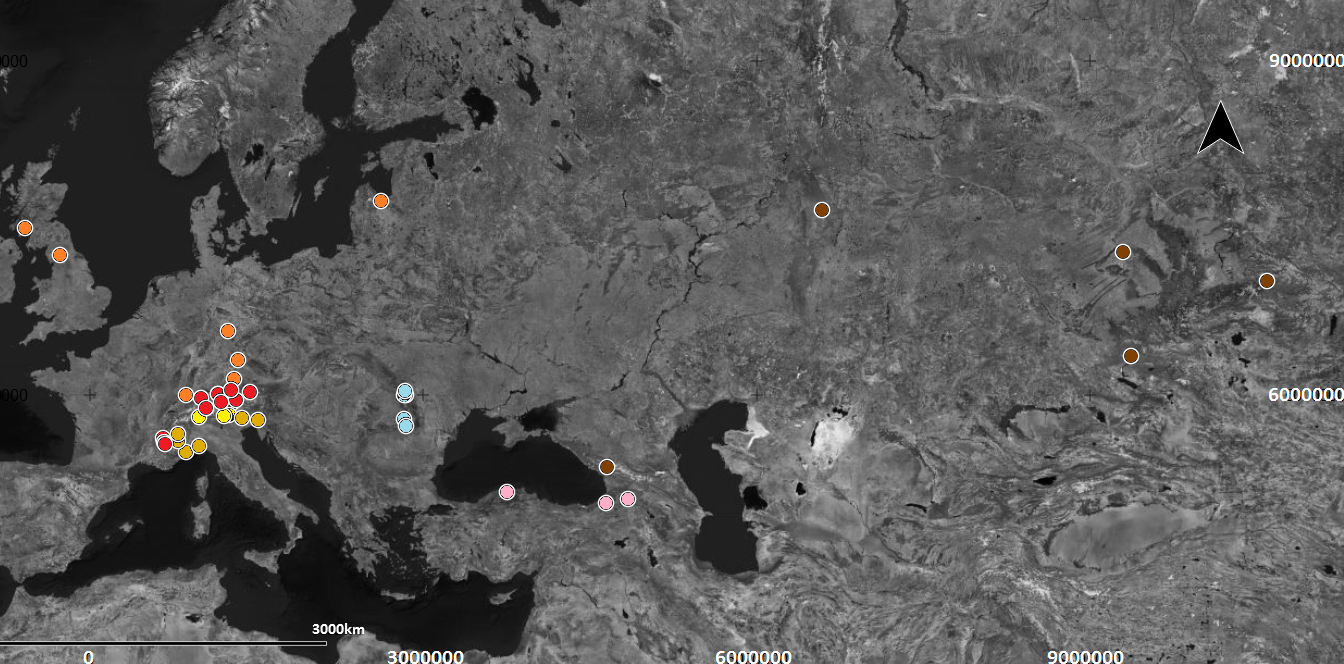


Fig. S16: Geographic location of the 48 COI sequence samples from BOLD and Genbank of *E. aethiops*. The geographical location of the haplotypes is given in same color scheme as in Fig. 4. The map was created with Qgis v.3.10.10^72^ (Available online: http://qgis.osgeo.org).

| Nucleotide sequence  CO1 | GenBank accession number | Nucleotide sequence  ND1 | GenBank accession number |
| --- | --- | --- | --- |
| BankIt2309627 H1 | MT017932 | BankIt2309627 H1 | MT017966 |
| BankIt2309627 H10 | MT017933 | BankIt2309627 H10 | MT017967 |
| BankIt2309627 H11 | MT017934 | BankIt2309627 H11 | MT017968 |
| BankIt2309627 H12 | MT017935 | BankIt2309627 H12 | MT017969 |
| BankIt2309627 H13 | MT017936 | BankIt2309627 H13 | MT017970 |
| BankIt2309627 H14 | MT017937 | BankIt2309627 H14 | MT017971 |
| BankIt2309627 H15 | MT017938 | BankIt2309627 H15 | MT017972 |
| BankIt2309627 H16 | MT017939 | BankIt2309627 H16 | MT017973 |
| BankIt2309627 H17 | MT017940 | BankIt2309627 H17 | MT017974 |
| BankIt2309627 H18 | MT017941 | BankIt2309627 H18 | MT017975 |
| BankIt2309627 H19 | MT017942 | BankIt2309627 H19 | MT017976 |
| BankIt2309627 H2 | MT017943 | BankIt2309627 H2 | MT017977 |
| BankIt2309627 H20 | MT017944 | BankIt2309627 H20 | MT017978 |
| BankIt2309627 H21 | MT017945 | BankIt2309627 H21 | MT017979 |
| BankIt2309627 H22 | MT017946 | BankIt2309627 H22 | MT017980 |
| BankIt2309627 H23 | MT017947 | BankIt2309627 H23 | MT017981 |
| BankIt2309627 H24 | MT017948 | BankIt2309627 H24 | MT017982 |
| BankIt2309627 H25 | MT017949 | BankIt2309627 H25 | MT017983 |
| BankIt2309627 H26 | MT017950 | BankIt2309627 H26 | MT017984 |
| BankIt2309627 H27 | MT017951 | BankIt2309627 H27 | MT017985 |
| BankIt2309627 H28 | MT017952 | BankIt2309627 H28 | MT017986 |
| BankIt2309627 H29 | MT017953 | BankIt2309627 H29 | MT017987 |
| BankIt2309627 H3 | MT017954 | BankIt2309627 H3 | MT017988 |
| BankIt2309627 H30 | MT017955 | BankIt2309627 H30 | MT017989 |
| BankIt2309627 H31 | MT017956 | BankIt2309627 H31 | MT017990 |
| BankIt2309627 H32 | MT017957 | BankIt2309627 H32 | MT017991 |
| BankIt2309627 H33 | MT017958 | BankIt2309627 H33 | MT017992 |
| BankIt2309627 H4 | MT017959 | BankIt2309627 H4 | MT017993 |
| BankIt2309627 H5 | MT017960 | BankIt2309627 H5 | MT017994 |
| BankIt2309627 H6 | MT017961 | BankIt2309627 H6 | MT017995 |
| BankIt2309627 H7 | MT017962 | BankIt2309627 H7 | MT017996 |
| BankIt2309627 H8 | MT017963 | BankIt2309627 H8 | MT017997 |
| BankIt2309627 H9 | MT017964 | BankIt2309627 H9 | MT017998 |
| BankIt2309627 EP_GF1 | MT017965 | BankIt2309627 EP_GF1 | MT017999 |
| *Pararge aegeria* | MH089839.1 | *Pararge aegeria* | KM592968.1 |

S17: Nucleotide sequences and GenBank accession numbers of *E. aethiops*; *E. pronoe* and *P. aegeria.*
